# Job strain and risk of obesity: systematic review and meta-analysis of cohort studies

**DOI:** 10.1038/ijo.2015.103

**Published:** 2015-06-30

**Authors:** M Kivimäki, A Singh-Manoux, S Nyberg, M Jokela, M Virtanen

**Affiliations:** 1 Department of Epidemiology and Public Health, University College London, London, UK; 2 Department of Public Health, Faculty of Medicine, University of Helsinki, Helsinki, Finland; 3 Inserm, Centre for Research in Epidemiology and Population Health, Hôpital Paul Brousse, Villejuif, France; 4 Finnish Institute of Occupational Health, Helsinki, Finland; 5 Institute of Behavioral Sciences, University of Helsinki, Helsinki, Finland

## Abstract

Job strain, the most widely used indicator of work stress, is a risk factor for obesity-related disorders such as cardiovascular disease and type 2 diabetes. However, the extent to which job strain is related to the development of obesity itself has not been systematically evaluated. We carried out a systematic review (PubMed and Embase until May 2014) and meta-analysis of cohort studies to address this issue. Eight studies that fulfilled inclusion criteria showed no overall association between job strain and the risk of weight gain (pooled odds ratio for job strain compared with no job strain 1.04, 95% confidence interval (CI) 0.99–1.09, *N*_Total_=18 240) or becoming obese (1.00, 95% CI 0.89–1.13, *N*_Total_=42 222). In addition, a reduction in job strain over time was not associated with lower obesity risk (1.13, 95% CI 0.90–1.41, *N*_Total_=6507). These longitudinal findings do not support the hypothesis that job strain is an important risk factor for obesity or a promising target for obesity prevention.

## Introduction

The obesity epidemic is a major public health challenge.^1^ Both physical inactivity and an unhealthy diet increase the risk of obesity, but interventions targeting these factors have not been successful in preventing or reversing obesity at the population level.^2, 3, 4^ Effective prevention, therefore, needs a strategy based on the importance of a wider range of risk factors.

Job strain, the most widely studied work-related psychosocial stressor, is seen as influencing the risk of obesity.^5, 6^ In agreement with this, meta-analyses have linked job strain to obesity in cross-sectional^7^ and to an increased risk of obesity-related disorders, such as type 2 diabetes, coronary heart disease and stroke in longitudinal studies.^8, 9, 10^ However, the extent to which job strain is related to weight gain and the development of obesity itself is poorly understood,^11, 12, 13, 14^ because quantitative reviews to confirm or refute these longitudinal associations are lacking. Similarly, it remains unclear whether a reduction in job strain is likely to reduce obesity risk and, thus, be a potentially useful target for obesity prevention.

Here we seek to address these questions by performing a systematic review and meta-analysis of prospective studies on the associations between job strain, change in job strain and the risk of weight gain and obesity.

## Methods

### Data sources and searches

Following the PRISMA guidelines,^15^ we performed a systematic search of the literature using ‘all fields' in PubMed and Embase on 21 August 2014; search terms: (job strain, job stress, work stress, job demands, job control, workload, job psychosocial or work psychosocial) and (body mass index, overweight, obesity or BMI). We also examined the reference lists of major reviews in the field^11, 12, 13, 14^ as well as those of the eligible publications, and performed a cited-reference search of these using the Institute of Scientific Information Web of Science (2014). Two authors (Kivimäki, Virtanen) independently reviewed titles and abstracts to retrieve potentially relevant studies and full articles were reviewed to evaluate whether they met the inclusion criteria.

### Inclusion criteria

We included studies that meet the following eligibility criteria: published in English; prospective design (cohort study) with individual level exposure and outcome data; examined the effect of job strain, as defined in the theoretical model by Karasek;^5^ weight gain or development of obesity as an outcome; and reported either estimates of relative risk, odds ratios or hazard ratios with 95% confidence intervals (CIs), or provided sufficient results to calculate these estimates.

### Data synthesis and statistical analysis

Meta-analysis was used to combine the estimates from the studies reported as odds ratios or relative risk. Associations of job strain with weight gain (yes/no) and becoming obese (yes/no) were analyzed separately. The basic model was adjusted for age, sex and socioeconomic status (SES) as covariates. SES is associated with job strain (the highest prevalence of job strain is typically in low-SES jobs) and multiple health outcomes, including obesity (overweight and obesity are most common in low-SES groups).^16, 17^ We also examined associations of increase and reduction in job strain and change in weight or obesity risk in studies where relevant data were available. We examined heterogeneity of study-specific estimates using the *I*^2^ statistic; as it was low (⩽48% in all analyses), we used fixed effects analysis (for comparison, results from random effects meta-analysis are reported in Supplementary Appendix). All statistical analyses were performed using Stata (MP version 11.2, College Station, TX, USA).

## Results

### Literature search of published studies

Initial search of PubMed and Embase retrieved 3579 results, but only eight cohort studies from five papers fulfilled inclusion criteria (for details, see Supplementary Appendix).^7, 18, 19, 20, 21^ By hand searching through reference lists within those studies, one additional study was identified.^22^ After removing one article reporting results from the same cohort,^21^ eight independent studies from five papers were included in the meta-analysis.^7, 18, 19, 20, 22^

### Meta-analysis of prospective cohort studies

**Figure 1:**
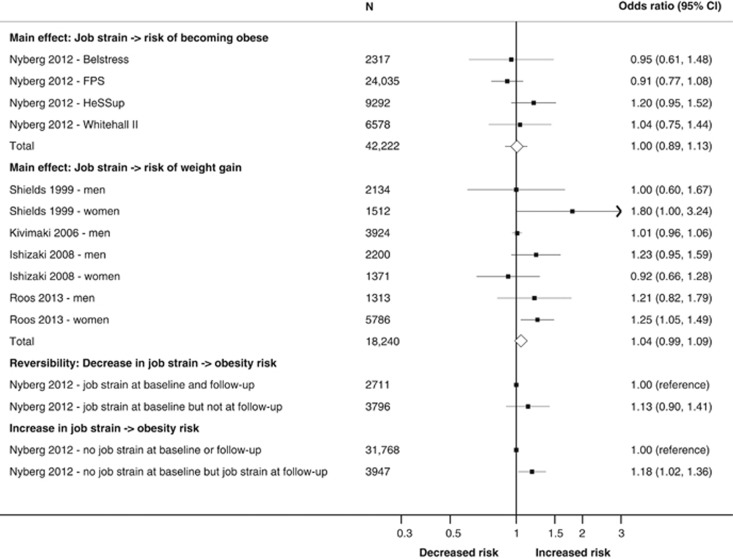
Meta-analysis of cohort studies on associations of job strain and change in job strain with risk of obesity and weight gain.

Four cohort studies contributed to the analysis of obesity^7^ but none reported a significant association between job strain and the risk of becoming obese (Figure 1). In fixed-effect meta-analysis, the minimally adjusted pooled odds ratio for job strain compared with no job strain was 1.00 (95% CI 0.89–1.13, total *N*=42 222) with little heterogeneity in study-specific estimates (*I*^2^=18.3%, *P*=0.30).^7^ Similarly, the pooled odds ratio did not show an association between job strain and gaining weight (1.04 (95% CI 0.99–1.09, total *N*=18,240, *I*^2^=48.4%, *P*=0.072),^18, 19, 20, 22^ although in two studies an association was observed in women (1.80, 95% CI 1.00–3.24 in Shields *et al.*^20^ and 1.25, 95% CI 1.05–1.49 in Roos *et al.*).^22^ The random effects modeling produced similar results. Moreover, the findings were not dependent on the length of follow-up time (data tabulated in the Supplementary Appendix).

None of these studies examined whether the association between job strain and risk of obesity or weight gain followed a dose–response gradient (that is, whether a greater exposure to job strain was associated with greater increases in obesity risk).

### Change in job strain in relation to change in obesity risk

Individuals who had job strain at baseline but not at follow-up did not have a lower risk of becoming obese compared with those who had job strain both at baseline and follow-up (pooled odds ratio 1.13, 95% CI 0.90–1.41, total *N*=6507).^7^ However, an increase in job strain was linked to increased obesity risk among initially job strain-free individuals, when compared with those with no persistent job strain (1.18, 95% CI 1.02–1.36, total *N*=35 715).^7^

## Discussion

In the studies identified from the literature, there was no overall association between job strain and risk of weight gain or obesity. An increase in job strain was linked to simultaneous increased obesity risk among initially job strain-free individuals, although a reduction in job strain, compared with persistent job strain, was not associated with reduced odds of obesity. These longitudinal findings on job strain and obesity are not consistent with the hypothesis that job strain is an important risk factor for obesity.

In prospective observational studies, demonstration of a temporal sequence in the exposure-outcome association supports the view that the exposure is a putative risk factor. Such evidence was not obtained for job strain as there was no overall association with weight gain and obesity. In the only statistically significant longitudinal observation, an increase in job strain between the baseline and follow-up was associated with a simultaneous increase in obesity.^7^ However, these contemporaneous changes did not allow us to determine whether the change in job strain preceded the change in obesity or whether weight gain increased subsequent risk of experiencing job strain.

Further considerations in determining whether an exposure is a genuine risk factor involve evidence of a graded dose–response association between an exposure and an outcome.^23^ Such pattern strengthens the evidence, although lack of dose response is not evidence against potential causal association because threshold effects are also possible.^23^ Dose response was examined only in one prospective study but that study did not fulfill the inclusion criteria for this meta-analysis.^21^ As the study used a modified iso-strain measure that includes social support in addition to job demands and job control, it is unclear whether the observed dose–response gradient also characterizes the job strain–obesity association. Cross-sectional data on job strain support this possibility but results from cross-sectional studies are known to be subject to reverse causation bias.^7^

The job strain model was originally developed for modeling psychosocial factors that affect mental health.^5^ Such problems, depression in particular, are by definition associated with an increase or decrease in appetite leading to weight gain in some patients and weight loss in others. It may therefore be hypothesized that job strain leads, directly or through increased mental health problems, to weight loss in some individuals, thus masking the overall association between job strain and weight gain.^18^ However, the evidence to support this possibility is inconsistent. In one study, job strain was associated with weight loss in men with low body mass index and weight gain in overweight and obese men, but no such weight changes were observed in women.^18^ Therefore, there is need for further research to determine the potential individual differences in response patterns when facing job strain and to examine the proportion of persons with increased unhealthy eating under stress, thus leading to an increased risk of gaining weight; those who lose appetite in such circumstances and are at an increased risk of weight loss; and those who remain unaffected in terms of eating behaviors, physical activity and the metabolic rate. However, given the lack of an association between job strain and risk of obesity in the total population, the first group is likely to be small.

Our meta-analysis benefits from focusing both on the presence of and change in job strain in relation to obesity risk. A drawback is that the assessment of job strain varied between the studies included in the analysis, which suggests that despite the concept of job strain concept being proposed already in 1979, there is still no general agreement on the best measure. Multiple and changing job strain definitions increase uncertainty in the results, making it impossible to determine whether the use of an alternative definition would have led to a different conclusion. Our evidence base of up to 44 000 individuals mainly consists of European studies and is much smaller than those reported in meta-analyses of job strain, coronary heart disease^9^ and type 2 diabetes.^8^ The generalizability of our findings should therefore be interpreted with caution, particularly as the statistical power was insufficient to detect small effects. Last, our meta-analysis was focused on one major work stressor, job strain, although other stressors (for example, effort-reward imbalance at work and care-giving burden in private life) might also be relevant. As job strain represents only a part of potential psychosocial stresses, we cannot exclude the possibility that prevention of stress in general, especially in combination with lifestyle interventions, could favorably affect weight management.

In conclusion, using prospective data, we show no consistent association between job strain and the development of obesity or weight gain. These findings suggest that job strain may not be an important risk factor for obesity and that job strain interventions alone, although improving psychological wellbeing at work, are not likely to alleviate the obesity epidemic, even in the working populations. However, because of the limitations in the published studies, we cannot exclude the possibility that there are subgroups of individuals who gain weight under job strain, although the null finding in the total data suggests that they represent the minority.

## Disclaimer

The funders had no role in the study design; in the collection, analysis and interpretation of data; in writing of the report; or in the decision to submit the paper for publication.
