# Supplementary material for: Job strain and risk of obesity: systematic review and meta-analysis of cohort studies
Source: Int J Obes (Lond). 2015 Jun 30;39(11):1597–600. doi: 10.1038/ijo.2015.103 (PMC4579559; doi:10.1038/ijo.2015.103)
Supplement: Supplementary Information [file ijo2015103x1.pdf]

Web Appendix: Kivimäki M, Singh-Manoux A, Nyberg S *et al*. Job strain and risk of obesity: Systematic review and meta-analysis of cohort studies. *Int J Obesity* 2015.

1. Selection of studies in the meta-analysis

Annex Figure 1. Flow diagram

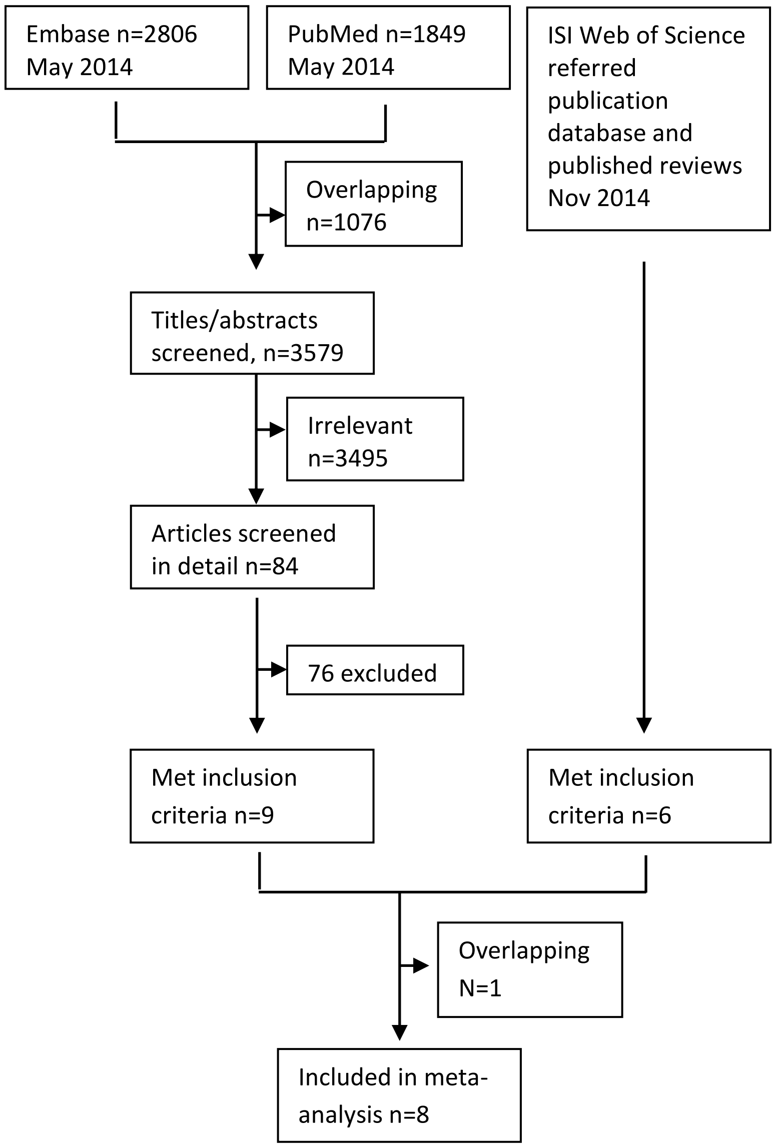

2. Random effects meta-analysis

The summary estimate for the association between job strain and the risk of becoming obese was very similar in the fixed effect modelling (1.00, 95% CI 0.89-1.13) and the random effects modelling (1.01, 95% CI 0.88-1.16, Annex Table 1). This was also the case for the association between job strain and subsequent weight gain (1.04, 95% CI 0.99-1.09 in the fixed effect modelling and 1.12, 95% CI 0.99-1.28 in the random effects modelling, Annex Table 2).

Annex Table 1. Random effect meta-analysis: Job strain -> becoming obese

Study	Follow-up time, years	Odds ratio (95% confidence interval)
Nyberg 2012 Belstress	6.5	0.95 (0.61 - 1.48)
Nyberg 2012 Finnish Public Sector Study	3.5	0.91 (0.77 - 1.08)
Nyberg 2012 HeSSup	5	1.20 (0.95 - 1.52)
Nyberg 2012 Whitehall II	5.5	1.04 (0.75 - 1.44)
Total		1.01 (0.88 - 1.16)

Heterogeneity chi-squared = 3.67 (d.f. = 3) p = 0.299

I-squared (variation in odds ratio attributable to heterogeneity) = 18.3%

Annex Table 2. Random effects meta-analysis: Job strain -> weight gain

Study	Follow-up time, years	Odds ratio (95% confidence interval)
Shields 1999 – men	2	1.00 (0.60 - 1.67)
Shields 1999 – women	2	1.80 (1.00 - 3.24)
Kivimäki 2006 – men	5.5	1.01 (0.96 - 1.06)
Ishizaki 2008 – men	6	1.23 (0.95 - 1.59)
Ishizaki 2008 –women	6	0.92 (0.66 – 1.28)
Roos 2013 – men	5-7	1.21 (0.82 – 1.79)
Roos 2013 – women	5-7	1.25 (1.05 – 1.49)
Total		1.12 (0.99 - 1.28)

Heterogeneity chi-squared = 11.63 (d.f. = 6) p = 0.071

I-squared (variation in odds ratio attributable to heterogeneity) = 48.4%

3. Meta-regression by length of follow-up

The follow-up time in studies of job strain and the risk of becoming obese varied between 3.5 and 6.5 years. Meta-regression suggests no statistically significant association between the length of follow-up time and effect size (i.e., the strength of the association between job strain and obesity risk)(Annex Table 3).

Annex Table 3. Meta regression: Effect of follow-up time on the association between job strain and obesity risk

	Effect size	
**Variable**	**Odds ratio (95% CI)**	**P-value**
Intercept	1.04 (0.72–1.48)	0.43
Follow-up time, year	1.06 (0.77–1.46)	0.76

Note. Follow-up intercept centered at 5 years.

The follow-up time in studies of job strain and weight gain varied between 2 and 7 years. Again meta-regression suggests no statistically significant association between the length of follow-up time and effect size (i.e., the strength of the association between job strain and the risk of weight gain)(Annex Table 4).

Annex Table 4. Meta regression: Effect of follow-up time on the association between job strain and risk of weight gain

	Effect size	
**Variable**	**Odds ratio (95% CI)**	**P-value**
Intercept	1.15 (0.95–1.38)	0.12
Follow-up time, year	0.96 (0.83–1.11)	0.50

Note. Follow-up intercept centered at 5 years.
